# Mobile point-of-care testing to improve access to care for people experiencing homelessness and diabetes in Los Angeles

**DOI:** 10.1093/ajcp/aqag107

**Published:** 2026-10-19

**Authors:** Tobin Berger-Cahn, Evan M Shannon, Brian Zunner-Keating, Catherine Weaver, Joshua Lee, Sae Takada, Cullen M Lilley, Imir G Metushi, Omai B Garner

**Affiliations:** David Geffen School of Medicine at UCLA, Los Angeles, CA, United States; Division of General Internal Medicine and Health Services Research, David Geffen School of Medicine at UCLA, Los Angeles, CA, United States; Homeless Healthcare Collaborative, UCLA Health, Los Angeles, CA, United States; Homeless Healthcare Collaborative, UCLA Health, Los Angeles, CA, United States; Department of Emergency Medicine, UCLA David Geffen School of Medicine, Los Angeles, CA, United States; David Geffen School of Medicine at UCLA, Los Angeles, CA, United States; Division of General Internal Medicine and Health Services Research, David Geffen School of Medicine at UCLA, Los Angeles, CA, United States; Department of Pathology and Laboratory Medicine, David Geffen School of Medicine at UCLA, Los Angeles, CA, United States; Department of Pathology and Laboratory Medicine, David Geffen School of Medicine at UCLA, Los Angeles, CA, United States; Department of Pathology and Laboratory Medicine, David Geffen School of Medicine at UCLA, Los Angeles, CA, United States

**Keywords:** point-of-care, diabetes, mobile clinic, homelessness, chronic disease, comprehensive metabolic panel, field medicine, mobile laboratory

## Abstract

**Objectives:**

To evaluate the utilization and results of point-of-care (POC) testing performed by the UCLA Health Homeless Healthcare Collaborative (HHC) to identify diagnostic patterns in patients experiencing homelessness (PEH) and specifically those with diabetes to inform future care models.

**Methods:**

A retrospective cohort study was performed using electronic health records of PEH who visited HHC mobile units between January 2023 and August 2024. Point-of-care test quantity, timing, and results were assessed. A comprehensive metabolic panel (CMP), blood glucose, and hemoglobin A1c (HbA1c) results were assessed for differences between diabetic and nondiabetic cohorts at initial presentation.

**Results:**

Between January 2023 and August 2024, the HHC served 4653 patients, with 836 receiving a POC test (17.97%). During this period, 126 basic metabolic panels, 197 CMPs, 102 COVID-19 antigen tests, 533 glucose tests, 166 HbA1c tests, 87 hemoglobin tests, 80 influenza A and B tests, 22 rapid strep A tests, 119 urinalysis dipstick tests, and 41 urine pregnancy tests were used. Of all tested, 190 (22.73%) received a diabetes *International Classification of Diseases* code. Within CMP results, mean sodium (139.5 vs 141.1 mmol/L; *P* < .05) and mean chloride (103.7 vs 105.8 mmol/L; *P* < .05) were both lower in patients with diabetes. The mean creatinine was not significantly different (0.91 vs 1.06 mg/dL; *P* = .097).

**Conclusions:**

Point-of-care testing provides critical diagnostic data for PEH, particularly those with diabetes, allowing for a clinician to initiate care prior to the development of severe disease. Expanding the test menu to include urinary albumin-to-creatinine ratio screening and creating POC-specific reference intervals is an opportunity for improvement.

Key points Point-of-care (POC) tests, including basic metabolic panel, comprehensive metabolic panel, glucose, COVID-19, influenza, strep, urinalysis, pregnancy, hemoglobin, and HbA1c, allow field-based homeless health care to diagnose and treat diseases among people experiencing homelessness.

1. People experiencing homelessness with diabetes present to care prior to the onset of severe renal disease detectable by serum creatinine measures.
2. People experiencing homelessness with diabetes make up a substantial portion of POC test utilization in a field-based health care system, and those with complications have significantly higher hemoglobin A1c measurements than those without.

## INTRODUCTION

The number of people experiencing homelessness (PEH) in the United States is at record-high levels. A point-in-time estimate conducted by the US Department of Housing and Urban Development predicted 771 480 PEH in 2024. A plurality of these individuals lived in California, where there were an estimated 187 084 PEH, and 66% of these individuals were unsheltered.^1^ It is more important than ever for health systems to assess their ability to serve this growing population of patients.

People experiencing homelessness experience stark disparities in chronic disease prevalence and comorbidity. One study of electronic health records (EHRs) in Ohio identified a greater prevalence of nearly every assessed condition when comparing PEH with housed patients.^2^ Chronic disease is also less likely to be adequately treated. People experiencing homelessness are approximately 3 times more likely to have cardiovascular disease and experience substantially increased mortality and morbidity.^3^^,^^4^ The prevalence of diabetes in PEH is believed to be similar to that of housed patients.^5^ Yet, there are higher rates of macrovascular complications from diabetes as well as hospitalization for hyperglycemia and infections.^6^ Those experiencing homelessness are not achieving adequate glycemic control and less frequently use primary care for diabetes management.^7^

People experiencing homelessness experience poor chronic disease control due to limited access to ambulatory care and a high prevalence of comorbid psychiatric, substance, and alcohol use disorders.^8‐10^ As a result, PEH disproportionately seek high-cost emergency care. Approximately 38% of California PEH reported an emergency department (ED) visit in the last 6 months in 2023 compared with approximately 20% of all adults surveyed by the National Health Interview.^11^^,^^12^ Furthermore, PEH who do present are substantially more likely to return to the ED in a short time frame.^13^

Because of the stark inequities faced by PEH, there is a need for the development of health care models specifically oriented to identify and manage chronic diseases among PEH before they require acute presentation to an ED.^8^ The UCLA Health Homeless Healthcare Collaborative (HHC) was launched in 2022 to address the growing population of PEH in Los Angeles County and their complex health needs. Health care workers in 6 mobile units meet patients where they are located for preventative health care visits, medication delivery, and chronic disease management. Each mobile unit is equipped with point-of-care (POC) tests. Central laboratory testing is not currently available for this population.

The goal of POC laboratory testing on a mobile unit is to maximize the number of high-quality and quickly resulting tests that are available while attempting to minimize the physical space that these tests and reagents take up in the mobile unit. While there are numerous feasible models for immediate testing and treatment of conditions such as hepatitis C and HIV,^14^^,^^15^ the development of a comprehensive POC test toolkit fit for the purpose of serving the full breadth of conditions experienced by PEH communities is still in progress. A preliminary 8-test POC menu was selected based on the most used tests in the outpatient UCLA Health setting and the existence of cost-effective laboratory instrumentation with feasible training requirements for clinical personnel. The POC testing included a basic metabolic panel (BMP), a comprehensive metabolic panel (CMP), hemoglobin A1c (HbA1c), urinalysis dipstick, hemoglobin, glucose, urine pregnancy, COVID-19 antigen testing, influenza A and B antigen testing, and group A strep testing.

Tests are selected based on clinical suspicion and diagnostic utility, as determined by the ordering clinician. For instance, a patient experiencing pharyngitis might receive a group A strep test dependent on clinical assessment. To ensure quality of testing, each test used in the mobile setting had a dedicated standard operating procedure, standardized training, and regular competency assessments for the users. This was modeled after the structure of POC testing within a clinical setting. Before testing began, the mobile units obtained a federal Clinical Laboratory Improvement Amendments Certificate of Waiver and a California State clinical laboratory registration from the California Department of Public Health. To our knowledge, the menu of POC tests offered by the HHC is one of the broadest among field-based homeless health care programs. Health systems looking to expand care in this space will benefit from seeing how our tests are used and with what results. Diabetes is a significant driver of health utilization in PEH and affects a substantial portion of our patients. We therefore focus on this diagnosis because it yields high-quality data and provides diagnostic context for PEH with a treatable condition prior to acute presentation to an ED, data that have been lacking in current literature.

Here we report the quantity, timing, and results of POC tests performed by HHC personnel and the demographics of patients receiving these tests. We also report differences in CMP, HbA1c, and blood glucose results between PEH with a diagnosis of diabetes and those without to inform how we may improve our care to these patients and identify key diagnostic considerations for clinicians caring for PEH. We hypothesize that PEH with a diagnosis of diabetes will demonstrate more frequent abnormalities in CMP results, including increased serum potassium and increased creatinine. We also hypothesize they will demonstrate higher HbA1c.

## METHODS

This study was reviewed and approved by the UCLA Institutional Review Board (IRB-23-1533). The EHRs of PEH who visited an HHC mobile clinic at least once were retrieved and hosted on a secure cloud-based computing environment called ULEAD. RStudio was used in this environment for all analysis and figure generation to ensure patient confidentiality and security.^16^

### Study design

Encounters, diagnoses, and laboratory tests ordered at HHC mobile clinics were filtered to those between January 1, 2023, and August 26, 2024. This date range was selected based on stable clinical volume 1 year after the start of HHC and the availability of data at the time of analysis. For count of tests performed by month, distinct combinations of result time, test description, and patient ID were blocked into monthly intervals, then grouped by test description and month interval and counted. For identification of total tests performed, distinct combinations of result time, test description, and patient ID were grouped by test description, diabetes status, and counted.

*International Classification of Diseases* (*ICD*) codes were assessed using the Clinical Classifications of Software Refined reference, which groups *ICD* codes into clinically meaningful conditions. Patient IDs were captured for tests ordered with a Clinical Classifications of Software Refined *ICD* group indication of either “diabetes mellitus with complication” or “diabetes mellitus without complication.”^17^ For the intent of analysis, these patient IDs were combined as diabetic or assessed individually as diabetic with and without complications. All other patients were defined as nondiabetic. This delineates those who received screening tests but never received a diabetic *ICD* code during the study period.

We assessed follow-up testing by counting, for HbA1c and glucose, the diabetic patients tested and those tested more than once (“retested”). We report the mean number of tests among retested patients. To explore glycemic monitoring, we present a table of the 20 diabetic patients with repeat POC HbA1c testing, listing their first and last values, the change between them, and the interval between these tests in days.

Diabetes with complications *ICD* codes present: E11.8, E11.42, E11.69, E11.65, E10.69, E11.59, E11.621, E11.52, E13.65, E11.39, E11.21, E11.40, E11.51, E11.49, E11.3552, E11.29, and E11.00. Diabetes without complications *ICD* codes present: E11.9, E10.9, and E13.9.

### Statistical analysis

Demographic characteristics were compared between PEH with and without diabetes. Median age and 25th to 75th percentile IQR are shown. Because age data were not assumed to follow a normal distribution, differences in age were assessed using the Wilcoxon rank-sum test. Sex was compared between groups using a Pearson χ^2^ test with Yates’ continuity correction. For total test count, we used a simple count of all tests during the study window, including serial testing if indicated in the figure legend. For test result analysis, the chronologically earliest combination of patient ID and test was retained to reduce the influence of serially tested individuals and ground the results in an identifiable point in time. Serial testing inclusion is noted in each figure and table legend for clarity. This represents the first patient presentation for the indicated laboratory test. Quantitative results were only assessed if they contained a numerical value. Several values were lost at this step because of potential error in data entry, result collection, or lack of sensitivity (ie, nonnumerical values such as HIGH or LOW were removed). Outliers beyond the 1st and 99th percentiles for each test were assessed for abnormalities. Two BMP results and 1 glucose result were removed due to being physiologically extremely unlikely. All other results are included in figures and analysis.

For both analysis and figure generation, CMP, HbA1c, blood glucose, and hemoglobin were divided by diabetic status. The decision was made to focus on CMP results for comparison due to the breadth of the analytes contained and broad use across both diabetic and nondiabetic groups. For continuous values, we calculated the mean result and its 95% confidence interval, estimated as mean ± 1.96 × SEM, where SEM = SD/√n. Mean results were compared using the *t* test. Normal laboratory ranges for men are indicated and sourced from the UCLA central clinical chemistry laboratory, apart from standard glucose, calcium, HbA1c, and hemoglobin, which had no population interval in our EHR at the time of data collection. Therefore, those ranges were sourced from the American College of Clinical Pharmacy.^18^ Statistical analyses were performed using t.test(), wilcox.test(), and chisq.test() functions in R.^19^ Figures for the quantitative results were created with ggplot2’s geom_dotplot function.^20^ Tables were generated using Microsoft Word.

### POC tests

Manufacturer and instrumentation for POC testing included Siemens Clinitek for urinalysis (10SG) and pregnancy; Quidel Sofia for COVID-19 antigen, influenza A and B antigen, and group A strep; and Hemocue glucose 201, Hemocue hemoglobin 201, Siemens DCA Vantage for Hemoglobin A1c, and Abaxis Piccolo Xpress for BMP and CMP.

## RESULTS

### Demographic and diabetic status of patients served by the HHC

During the study period, 4653 total patients were served by the HHC. This cumulative patient population had a median age of 48 (IQR, 37-59) and was predominantly male (66.45%), with a demographic breakdown of 20.2% Hispanic (all races), 12.36% non-Hispanic Black, 13.02% non-Hispanic White, and 54.42% other/unknown. Of 4653 total patients, 836 received a POC test (17.97%). The tested patient population had a median age of 53 (IQR, 42-62) years and was predominantly male (61.48%). Of 836 tested patients, 190 (22.73%) received a diabetes *ICD* code. People experiencing homelessness with diabetes had a significantly higher median age of 58 (IQR, 49-64) years vs 52 (IQR, 40-61) years by the Wilcoxon rank-sum test (W: 46 682, *P* < .001) and were predominantly male (76.84% vs 56.97%, χ² = 23.66, *df* = 1, *P* < .001) (Table 1). Within that diabetic group, 72 (37.89%) patients were given a diabetes with complications *ICD* code.

**Table 1:** Demographics of Patients Encountered Between January 1, 2023, and August 26, 2024

ICD group	No.	Age, median (IQR), y	Male, No. (%)	Female, No. (%)	Hispanic (all races), No. (%)	Non-Hispanic Black, No. (%)	Non-Hispanic White, No. (%)	Other/unknown, No. (%)
All	4653	48 (37-59)	3092 (66.45)	1559 (33.51)	940 (20.20)	575 (12.36)	606 (13.02)	2532 (54.42)
All tested	836	53 (42-62)	514 (61.48)	322 (38.52)	205 (24.52)	97 (11.60)	95 (11.36)	439 (52.51)
Nondiabetic	646	52 (40-61)	368 (56.97)	278 (43.03)	148 (22.91)	78 (12.07)	75 (11.61)	345 (53.41)
Diabetic	190	58 (49-64)	146 (76.84)	44 (23.16)	57 (30.00)	19 (10.00)	20 (10.53)	94 (49.47)
Diabetic without complications	118	57 (50-64)	93 (78.81)	25 (21.19)	30 (25.42)	11 (9.32)	11 (9.32)	66 (55.93)
Diabetic with complications	72	60 (49-65)	53 (73.61)	19 (26.39)	27 (37.50)	8 (11.11)	9 (12.50)	28 (38.89)

Abbreviation: ICD, International Classification of Diseases.

### Monthly and total POC test utilization

The aggregate number of each test per month over the study period is shown in Figure 1. Utilization of CMP, BMP, and HbA1c increased over time, while COVID-19 antigen, hemoglobin, urinalysis dipstick, urine pregnancy, glucose, and rapid strep A remained stable over time. Influenza A and B testing showed a seasonal pattern, with peaks from November through January (Figure 1A). During the study period, 126 BMPs, 197 CMPs, 102 COVID-19 antigen tests, 533 glucose tests, 166 HbA1c tests, 87 hemoglobin tests, 80 influenza A and B tests, 22 rapid strep A tests, 119 urinalysis dipstick tests, and 41 urine pregnancy tests were performed (Table 2).

**Figure 1:**
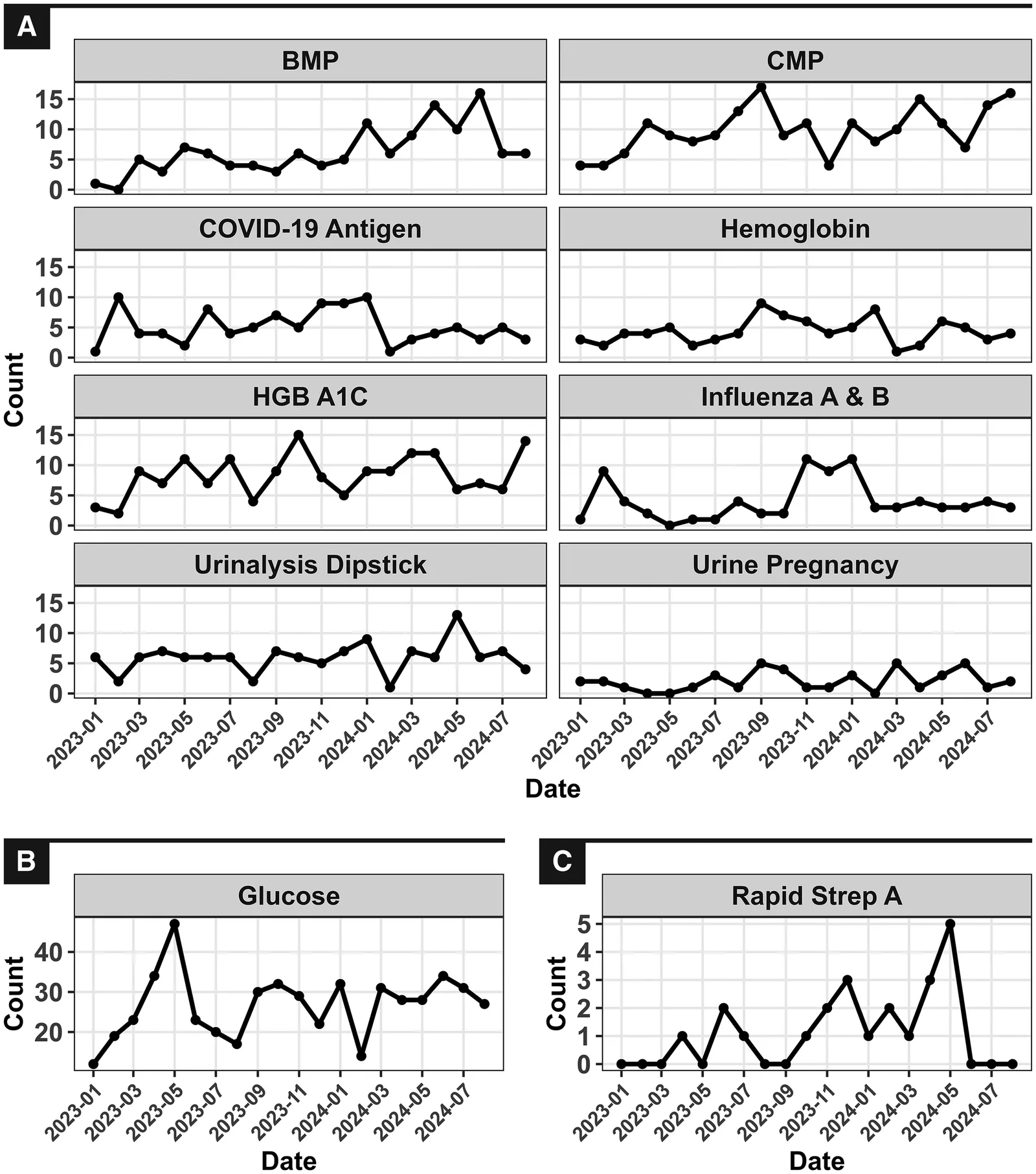
Monthly point-of-care test volumes from January 1, 2023, to August 26, 2024. Test count includes serial testing. (A) Basic metabolic panel (BMP), comprehensive metabolic panel (CMP), COVID-19 antigen, hemoglobin, hemoglobin A1c (HbA1c), influenza A and B, urinalysis dipstick, urine pregnancy. (B) Individual glucose test. (C) Rapid strep A.

**Table 2:** Number of Tests Performed for All Patients and Nondiabetic and Diabetic Fractions Determined by *International Classification of Diseases* Code^a^

Laboratory test	All tested, No.	Nondiabetic, No. (% all tested)	Diabetic, No. (% all tested)
BMP	126	84 (66.67)	42 (33.33)
CMP	197	157 (79.70)	40 (20.30)
COVID-19	102	94 (92.16)	8 (7.84)
Glucose	533	263 (49.34)	270 (50.66)
HbA1c	166	40 (24.10)	126 (75.90)
Hemoglobin	87	82 (94.25)	5 (5.75)
Influenza	80	72 (90.00)	8 (10.00)
Rapid strep A	22	22 (100.00)	0 (0.00)
Urinalysis	119	107 (89.92)	12 (10.08)
Pregnancy	41	41 (100.00)	0 (0.00)

Abbreviations: BMP, basic metabolic panel; CMP, comprehensive metabolic panel; HbA1c, hemoglobin A1c.

a Test count includes serial testing.

### Urinalysis dipstick distribution of results

Urinalysis dipstick testing revealed 13.2% glucose positive and 9.4% with glucosuria more than 1000 mg/dL. Bilirubin positivity was 17.1%, with 1.9% being moderate/large findings. Overall ketone positivity was 16.2%, with 4.8% being moderate/large. Blood positivity was 42.5%, with 20.8% moderate/large. Protein positivity was 57.1%, with 4.8% being more than 300 mg/dL. Nitrate positivity was 17.1%. Leukocyte esterase positivity was 42.9%, with 29.5% being moderate/large. For specific gravity, 1.9% were low (<1.005), and 1% were high (>1.030). For pH, 24.8% were acidic (pH <5.0), and 2.9% were alkaline (pH >8.0). For urobilinogen, 1% was abnormally high (>1 mg/dL) (Table 3).

**Table 3:** Semiquantitative Results for Point-of-Care Urinalysis Dipstick Tests Between January 1, 2023, and August 26, 2024^a^

Analyte	No.	Negative, No. (%)	Any positive, No. (%)	Key high-grade abnormal, No. (%)	
Glucose	106	92 (86.8)	14 (13.2)	1000 mg/dL: 10 (9.4)	
Bilirubin	105	87 (82.9)	18 (17.1)	Moderate/large: 2 (1.9)	
Ketones	105	88 (83.8)	17 (16.2)	Moderate/large: 5 (4.8)	
Blood	106	61 (57.5)	45 (42.5)	Moderate/large: 22 (20.8)	
Protein	105	45 (42.9)	60 (57.1)	≥300 mg/dL: 5 (4.8)	
Nitrate	105	87 (82.9)	18 (17.1)	—	
Leukocyte esterase	105	60 (57.1)	45 (42.9)	Moderate/large: 31 (29.5)	
Specific gravity	105			Low <1.005: 2 (1.9) High >1.030: 1 (1)	
pH	105			Acidic ≤5.0: 26 (24.8) Alkaline ≥8.0: 3 (2.9)	
Urobilinogen	105			Abnormal >1 mg/dL: 1 (1)	

a Results are the first to appear in the study window and exclude serial testing.

### COVID-19, influenza A and B, rapid strep A, urine pregnancy, and hemoglobin distribution of results

For infectious clinical indications, COVID-19 antigen, influenza A and B, and rapid strep A tests are provided to HHC personnel. Over the study period, 9 of 101 COVID-19 antigen results were positive (8.9%). Of 21 rapid strep A results, 3 (14.3%) were positive. For gynecologic indications, a urine pregnancy test is provided; among 37 tests performed, 2 (5.4%) were positive. For influenza A, 1 of 80 tests (1.3%) was positive; for influenza B, 9 of 80 tests (11.3%) were positive. To assess for anemia, a hemoglobin test is provided. The mean hemoglobin for nondiabetic patients was 11.87 (n = 75; 95% CI, 11.31-12.43) (Table 4). Hemoglobin tests were primarily used for patients with hematologic complaints such as aplastic anemia or fatigue (data not shown). They were infrequently used for PEH with diabetes.

**Table 4:** Summary of Results for Blood Glucose, HbA1c, and Hemoglobin Tests Between January 1, 2023, and August 26, 2024^a^

Test	Diabetic, mean (n, 95% CI)	Nondiabetic, mean (n, 95% CI)	*P* value
Glucose, mg/dL	215.18 (148, 198.67-231.69)	129.10 (234, 122.26-135.94)	<.001
HbA1c, %	8.35 (97, 7.86-8.85)	5.66 (40, 5.25-6.06)	<.001
Hemoglobin, g/dL	13.04 (5)	11.87 (75, 11.31-12.43)	
**Test**	**Complicated, mean (n, 95% CI)**	**Uncomplicated, mean (n, 95% CI)**	***P* value**
Glucose, mg/dL	237.36 (60, 210.92-263.81)	202.67 (88, 181.82-223.52)	.077
HbA1c, %	8.99 (43, 8.25-9.72)	7.85 (54, 7.21-8.49)	.024
Hemoglobin, g/dL	11.75 (2)	13.90 (3)	

Abbreviation: HbA1c, hemoglobin A1c.

a Results are the first to appear in the study window and exclude serial testing. Results were first divided by diabetic and nondiabetic status for analysis, then by diabetes with complications and without. *P* value calculated using the *t* test.

### Glucose and HbA1c distribution of results for patients with and without diabetes

For diabetes screening and blood sugar management, blood glucose and HbA1c tests are provided. At first presentation, the mean blood glucose result for PEH with diabetes was significantly higher than for those without (215.18 vs 129.1 mg/dL; *P* < .001). The mean HbA1c was also significantly higher (8.35 vs 5.66%; *P* < .001). However, on first measurement, only 41.2% of patients with diabetes had HbA1c test results less than 7%. When separating diabetic patients with and without complications, we found that patients with complications had a significantly higher HbA1c (8.99 vs 7.85%; *P* = .024). There was no significant difference in blood glucose results. (Table 4).

### Glucose and HbA1c follow-up testing for patients with diabetes

Patients with diabetes often receive serial blood glucose monitoring and HbA1c measurement. We found that 148 PEH with diabetes received a blood glucose test, and 54 were retested for blood glucose (36.5%). On average, 3.26 blood glucose tests were used per retested patient. Of 97 PEH with diabetes who received an HbA1c measurement, 20 were retested (20.6%). The average number of HbA1c measurements for those retested was 2.45. Among these patients, 8 had a decline between the last and first HbA1c measurements (40%) (Table 5).

**Table 5:** Longitudinal HbA1c Results in Diabetic Patients With Repeat Testing Between January 1, 2023, and August 26, 2024^a^

Patient	No. of tests	First HbA1c	Last HbA1c	Change	Interval, d
1	2	10.8	7.5	–3.3	268
2	2	13	10.5	–2.5	56
3	2	7.9	5.9	–2	140
4	2	9.3	7.6	–1.7	392
5	2	9.3	7.7	–1.6	252
6	4	7.8	6.3	–1.5	154
7	3	6.2	5	–1.2	329
8	2	5.8	5.6	–0.2	112
9	2	6	6	0	91
10	2	5.6	5.7	0.1	311
11	2	5.9	6.1	0.2	97
12	3	5.7	6.2	0.5	224
13	4	6	6.5	0.5	291
14	3	11.9	12.5	0.6	259
15	2	6.3	7	0.7	63
16	3	6.1	6.8	0.7	322
17	2	8.2	8.9	0.7	168
18	3	7.7	8.7	1	196
19	2	10.5	13	2.5	224
20	2	6.9	11	4.1	188

Abbreviation: HbA1c, hemoglobin A1c.

a Tests indicate the number of HbA1c tests performed per patient. First HbA1c and last HbA1c denote the earliest and most recent values recorded. Interval in days between the first and last test is provided. Change is the difference between the last and first measurements. Ordered by magnitude of change.

### CMP distribution of results for patients with and without diabetes

A CMP test on HHC mobile units contained values for sodium, potassium, urea nitrogen, creatinine, chloride, total CO_2_, glucose, calcium, alanine aminotransferase, aspartate aminotransferase (AST), alkaline phosphatase, albumin, total protein, and total bilirubin, which allows for assessment of renal, hepatic, metabolic, and electrolyte disorders. The mean sodium result for PEH with diabetes was slightly lower than those with no diabetes diagnosis (139.49 vs 141.1 mmol/L; *P* = .047). The mean potassium result for PEH with diabetes was not significantly greater than those without (4.4 vs 4.3 mmol/L; 95%; *P* = .212). The mean creatinine result for PEH with diabetes was not significantly different from those without (0.91 vs 1.06 mg/dL; *P* = .097). The mean chloride result for PEH with diabetes was significantly lower than those without (103.7 vs 105.8 mmol/L), and both distributions were close to the upper limit of most clinical laboratories (*P* = .015). The mean glucose result for PEH with diabetes was significantly higher than those without (256.6 vs 120.2 mg/dL; *P* < .001). The mean AST result for PEH with diabetes was significantly lower than those without (30 vs 40.9 U/L; *P* < .001). The mean albumin result for PEH with diabetes was not significantly lower than those without (3.5 vs 3.6 g/dL), and both distributions were near the lower limit of most clinical laboratories (*P* = .798). For complete tabulated results, see Table 6 and Figure 2.

**Figure 2:**
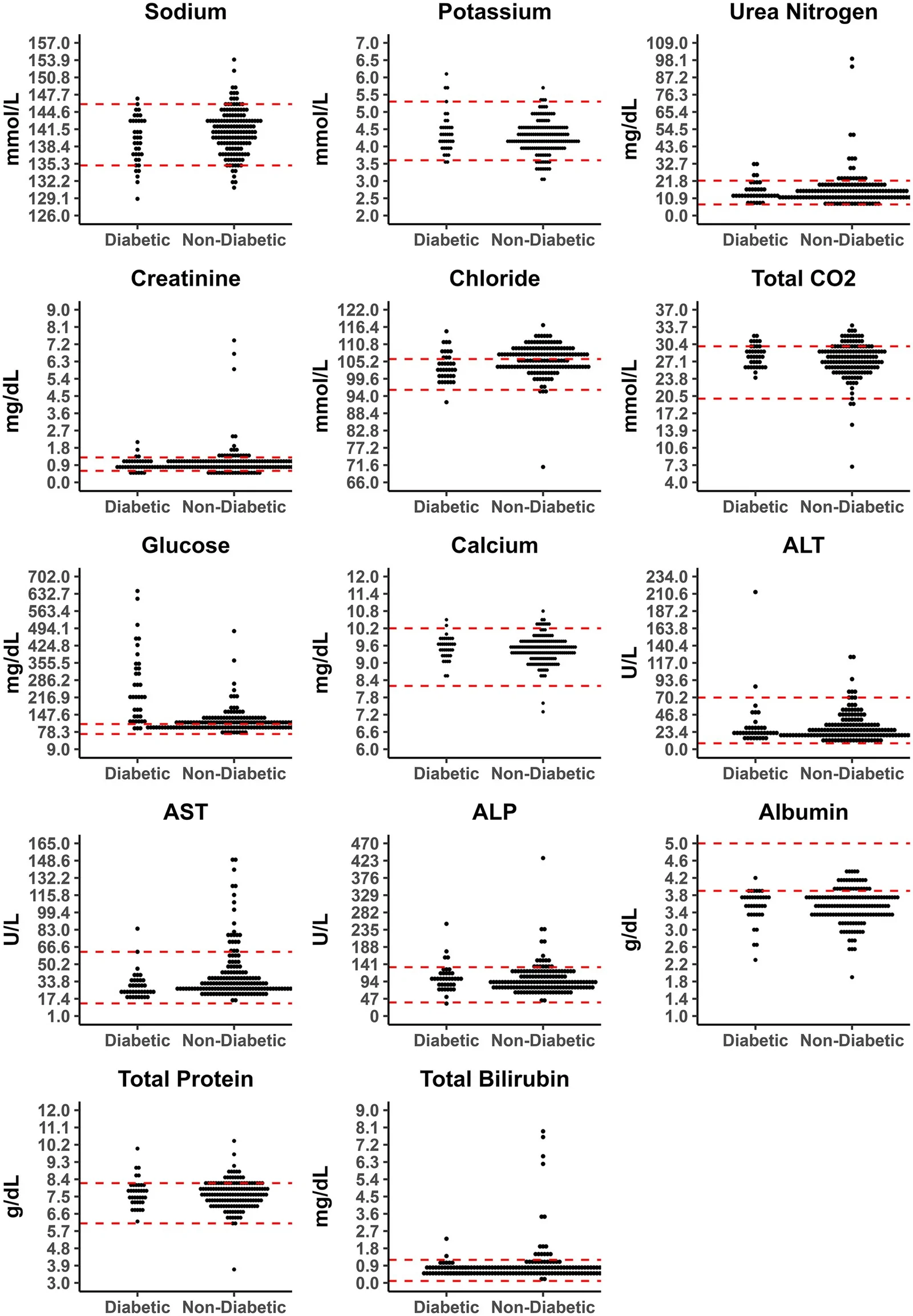
Distribution of results for comprehensive metabolic panel tests between January 1, 2023, and August 26, 2024. Results are the first to appear in the study window and exclude serial testing. Results are divided by diabetic and nondiabetic status. Clinical reference ranges are indicated with a red dotted line.

**Table 6:** Summary of Results for CMP Tests Between January 1, 2023, and August 26, 2024^a^

CMP	Reference range	Diabetic, mean (n, 95% CI)	Nondiabetic, mean (n, 95% CI)	P value
Sodium, mmol/L	135-146	139.49 (37, 138.1-140.87)	141.07 (150, 140.44-141.69)	.047
Potassium, mmol/L	3.6-5.3	4.4 (37, 4.21-4.59)	4.27 (150, 4.19-4.35)	.212
Urea nitrogen, mg/dL	7-22	15.22 (37, 13.19-17.24)	16.33 (149, 14.46-18.2)	.431
Creatinine, mg/dL	0.6-1.3	0.91 (37, 0.8-1.02)	1.06 (149, 0.92-1.2)	.097
Chloride, mmol/L	96-106	103.65 (37, 102.16-105.14)	105.8 (149, 105.01-106.59)	.015
Total CO_2_, mmol/L	20-30	28.22 (37, 27.56-28.87)	27.39 (150, 26.85-27.93)	.059
Glucose, mg/dL	70-110	256.57 (37, 209.37-303.77)	120.15 (150, 112.53-127.76)	<.001
Calcium, mg/dL	8.2-10.2	9.53 (37, 9.4-9.66)	9.46 (150, 9.38-9.53)	.357
Alanine aminotransferase, U/L	8-70	32.03 (37, 21.1-42.95)	30.47 (150, 27.35-33.59)	.789
Aspartate aminotransferase, U/L	13-62	30 (37, 25.87-34.13)	40.93 (150, 36.81-45.04)	<.001
Alkaline phosphatase, U/L	37-113	104.35 (37, 92.14-116.56)	100.43 (150, 93.81-107.04)	.582
Albumin, g/dL	3.9-5	3.54 (37, 3.41-3.67)	3.56 (150, 3.49-3.62)	.798
Total protein, g/dL	6.1-8.2	7.68 (37, 7.44-7.91)	7.61 (150, 7.49-7.73)	.616
Total bilirubin, mg/dL	0.1-1.2	0.76 (37, 0.65-0.87)	0.94 (149, 0.76-1.12)	.110

Abbreviation: CMP, comprehensive metabolic panel.

a Results are the first to appear in the study window and exclude serial testing. Results are divided by diabetic and nondiabetic status. Laboratory reference ranges for men are sourced from the UCLA Central Clinical Chemistry Lab, apart from calcium and glucose, which are sourced from the American College of Clinical Pharmacy. *P* value calculated using the *t* test.

### POC test utilization for patients with and without diabetes

There were clear differences in test utilization between patients with and without a diagnosis of diabetes (Table 2). Despite fewer patients having diabetes (n = 190 vs 646), half of glucose tests administered were to patients with diabetes (n = 270 vs 263; 50.7% diabetic tests). Furthermore, HbA1c testing was primarily performed for patients with diabetes (n = 126 vs 40; 75.9% diabetic tests). Basic metabolic panels were used less often for patients with diabetes (n = 42 vs 84; 33.3% diabetic), as were CMPs (n = 40 vs 157; 20.3% diabetic). Patients with diabetes also received fewer urinalysis tests (n = 12 vs 107; 10.1% diabetic).

## DISCUSSION

Our study at a field-based homeless health care program in Los Angeles County found that 836 of 4653 (18%) patients served received POC testing. We found that blood glucose, CMP, HbA1c, and BMP tests were administered most frequently. Overall, mean hemoglobin and albumin were low, and urinalysis had a 17% nitrate positivity rate. Stratifying patients by diabetes diagnosis, blood glucose, and HbA1c was performed more often, and urinalysis was performed less often for patients with diabetes. Still, approximately half of the blood glucose tests were used for patients without diabetes, which suggests its continued use as a screening tool for hypoglycemia, diabetic ketoacidosis, or hyperglycemic state. Patients with diabetes had higher serum glucose and lower serum sodium and chloride, lower AST, and similar serum creatinine compared with patients without diabetes. Despite rapid adaptation of POC equipment, test utilization has increased slowly and heterogeneously. There was immediate uptake of influenza and COVID-19 tests, which are traditional lateral-flow POC platforms. The otherwise slow rise in utilization of tests such as CMP and BMP may be driven by increasing staff training and comfort with more complicated assay platforms.

Compared with the central laboratory reference interval, which populates in our EHR, CMP albumin values were almost uniformly low (Table 6 and Figure 2). To evaluate this discrepancy, we identified the manufacturer package insert reference range. For PEH, of 197 CMP albumin values, 159 fall below the 3.9-g/dL central laboratory value, and 32 values fall below the 3.3-g/dL manufacturer value.^21^ In practice, when results are clearly abnormal and match the clinical picture, modest discrepancies in reference intervals may not change management. For instance, we identified 4 patients with hemoglobin levels below 7, and within a month, most received prescriptions for iron supplementation, with 1 presenting to the ED for blood transfusion (data not shown). However, in a population with a high prevalence of chronic disease, detecting those affected by indolent pathology is important. Serum albumin has high prognostic value for morbidity and mortality across a broad range of diseases. Inflammation causes increased vascular permeability to proteins and increased interstitial space for their accumulation, leading to hypoalbuminemia.^22^ Albumin may be lost in renal pathology such as nephrotic syndrome or be lower due to hepatic dysfunction, where these proteins are created.^23^ Serum albumin remains reliable even in the context of malnutrition, as changes typically only occur in terminal stages of starvation.^23^

It is not possible to definitively determine the normal distribution of albumin results in this study, but given our study population, we believe there is likely a component of true hypoalbuminemia as well as inadequate reference range selection. We therefore believe an HHC clinician is unlikely to recognize mild albumin loss, which may delay identification of evolving pathology. To mitigate reference interval discrepancies, each clinical laboratory should ideally establish its own normal reference intervals based on healthy individuals for each test in use. Standard practice is to sample at least 120 healthy patients from each relevant age group, sex, and subpopulation to define the central 95% of observed values as the reference interval.^24^ Even when the best practices are followed, reference ranges can vary widely across laboratories. As a result, clinicians using POC testing may use reference ranges to guide care that is not fully aligned with either the population or the specific instrument used.

When stratifying by diabetic status, there were some notable differences between patient groups. Compared with patients without diabetes, patients with diabetes had lower serum sodium (139.5 vs 141.1 mmol/L) and chloride mean (103.7 vs 105.8 mmol/L). These results are consistent with physiologic hyponatremia due to water shifting from the intracellular to the extracellular space in response to hyperglycemia.^25^^,^^26^ Serum potassium is typically elevated for patients with diabetes^26^; however, in our study, potassium was not significantly higher. Interestingly, the mean creatinine result for patients with diabetes was not significantly different. Early microvascular damage to the kidney among patients with diabetes might be better detected by monitoring urine albumin and creatinine. The American Diabetes Association (ADA) recommends a spot urinary albumin-to-creatinine ratio test.^27^ This test is available as a POC test and may be an important addition to screening for microvascular disease among PEH with diabetes.

Urinalysis was performed less often in the diabetic group than in the nondiabetic group. The number of urinalysis tests demonstrating glucosuria was also consistent with the number of patients with diabetes tested (Table 2 and Table 3). We primarily performed HbA1c testing in our cohort of patients with diabetes, and the initial mean HbA1c was elevated at 8.35% (95% CI, 7.86-8.85) compared with a typical goal for adults with diabetes of less than 7%. Interestingly, 41.2% of these patients had HbA1c results less than 7%, suggesting that a subgroup of these patients experienced worse glycemic control. When stratifying by *ICD* codes designating a complication from diabetes, we found that those with complications had a significantly higher HbA1c than those without (8.99 vs 7.85; *P* = .024) (Table 4). The group of PEH presenting with HbA1c at goal may have received diabetes care elsewhere.

Despite that, most HbA1c measurements were still not at goal, and patients with and without complications maintained similarly high blood glucose measurements. Our POC platforms have allowed us to follow up with many of these patients. Of PEH with diabetes who received a blood glucose test, 36.5% were retested. For HbA1c testing, 20.6% were retested. Of those 20 patients who received serial HbA1c measurements, 40% exhibited a decreased level from first to last measurement. Measurements were taken between 56 and 392 days apart (Table 5). Furthermore, on examining records from patients with very high HbA1c more than 11%, most were provided prescriptions for antidiabetic medications within a month (data not shown). These platforms allow our clinicians to immediately begin treatment for PEH with diabetes and trend longitudinal glycemic data in a field-based setting when many of these patients cannot access a central laboratory.

Given their large population, special consideration should be given to patients with diabetes when planning future additions to a POC testing menu. The primary therapeutic goal in diabetes management is to achieve and maintain glycemic control to reduce the risk of complications from prolonged hyperglycemia. Existing POC assays for glucose, CMP, BMP, and HbA1c are likely sufficient for routine monitoring of glycemic control and later stages of disease progression. However, the ADA recommends earlier surveillance for end-organ damage to prevent severe complications such as chronic kidney disease and blindness secondary to diabetic retinopathy.^27^

This study has several important limitations. First, because *ICD* codes are assigned when tests are ordered or entered into the EHR, it is not always possible to distinguish between clinical suspicion and laboratory diagnosis. However, in our system, these *ICD* codes often align with reported laboratory results. Second, many tests are used predominantly in one group or the other, which limits direct comparison between patients with and without diabetes. It is difficult to infer why tests were selected for one group based solely on diagnosis at the time of ordering. Third, we account for serial testing only in usage and follow-up testing data but not in result analysis. Fourth, general reference intervals are provided from our core clinical laboratory and EHR when available but are not generated for the specific population and instrument used. Finally, the EHR data are often incomplete; for example, race and ethnicity are missing for most patients, which constrains our ability to assess disparities and adjust for demographic factors.

The findings of this study suggest that mobile laboratory platforms can reach PEH with chronic disease and provide diagnostic context to facilitate immediate care. Furthermore, patients with diabetes are encountered prior to the onset of severe manifestations of diabetic kidney disease. Care of these patients may be improved by the addition of a POC spot urinary albumin-to-creatinine ratio, which would allow for proactive renal monitoring and initiation of renal protective therapies. Finally, this study emphasizes a need to prioritize more relevant reference intervals in a POC context. Improved knowledge of test parameters is an opportunity to improve care, as it allows identification of more subtle disease development.

## Data Availability

Data are not publicly available to protect patient confidentiality but may be provided on a secure platform pending approval of a UCLA CTSI data release request.
